# Management and prevention of anemia (acute bleeding excluded) in adult critical care patients

**DOI:** 10.1186/s13613-020-00711-6

**Published:** 2020-07-22

**Authors:** Sigismond Lasocki, Frédéric Pène, Hafid Ait-Oufella, Cécile Aubron, Sylvain Ausset, Pierre Buffet, Olivier Huet, Yoann Launey, Matthieu Legrand, Thomas Lescot, Armand Mekontso Dessap, Michael Piagnerelli, Hervé Quintard, Lionel Velly, Antoine Kimmoun, Gérald Chanques

**Affiliations:** 1 Département d’anesthésie-réanimation, Pôle ASUR, CHU Angers, UMR INSERM 1084, CNRS 6214, Université d’Angers, 49000 Angers, France; 2 Service de Médecine Intensive et Réanimation, Hôpital Cochin, Assistance Publique-Hôpitaux de Paris. Centre, Université de Paris, Paris, France; 3 Service de Médecine Intensive et Réanimation, Hôpital Saint-Antoine, Assistance Publique-Hôpitaux de Paris, Université Pierre et Marie Curie Paris, Paris, France; 4 Médecine Intensive Réanimation, CHRU de Brest, Université de Bretagne Occidentale, 29200 Brest, France; 5 Ecoles Militaires de Santé de Lyon-Bron, 69500 Bron, France; 6 Université de Paris, UMRS 1134, Inserm, 75015 Paris, France; 7 Laboratory of Excellence GREx, 75015 Paris, France; 8 Département d’Anesthésie Réanimation, Hôpital de la Cavale-Blanche, CHRU de Brest, 29200 Brest, France; 9 UFR de Médecine de Brest, Université de Bretagne Occidentale, 29200 Brest, France; 10 Critical Care Unit, Department of Anaesthesia, Critical Care Medicine and Perioperative Medicine, Rennes University Hospital, 2, Rue Henri-Le-Guilloux, 35033 Rennes, France; 11 Department of Anaesthesiology and Perioperative Care, University of California San Francisco, San Francisco, CA USA; 12 Département d’Anesthésie-Réanimation, Hôpital Saint-Antoine, Sorbonne Université, Assistance Publique-Hôpitaux de Paris, Paris, France; 13 AP-HP, Hôpitaux Universitaires Henri-Mondor, DMU Médecine, Service de Médecine Intensive Réanimation, 94010 Créteil, France; 14 Intensive Care, CHU-Charleroi Marie-Curie, Experimental Medicine Laboratory, Université Libre de Bruxelles, (ULB 222) Unit, 140, Chaussée de Bruxelles, 6042 Charleroi, Belgium; 15 Réanimation Médico-Chirurgicale, Hôpital Pasteur 2, CHU Nice, 30, Voie Romaine, Nice, France; 16 AP-HM, Department of Anaesthesiology and Critical Care Medicine, University Hospital Timone, 13005 Marseille, France; 17 Aix Marseille University, CNRS, Inst Neurosci Timone, UMR7289, Marseille, France; 18 Service de Médecine Intensive et Réanimation Brabois, Université de Lorraine, CHRU de Nancy, Inserm U1116, Nancy, France; 19 Department of Anaesthesia and Intensive Care, Montpellier University Saint-Eloi Hospital, and PhyMedExp, INSERM, CNRS, University of Montpellier, Montpellier, France

**Keywords:** Guidelines, Anemia, Blood transfusion, Erythropoietin, Iron

## Abstract

**Objective:**

Anemia is very common in critical care patients, on admission (affecting about two-thirds of patients), but also during and after their stay, due to repeated blood loss, the effects of inflammation on erythropoiesis, a decreased red blood cell life span, and haemodilution. Anemia is associated with severity of illness and length of stay.

**Methods:**

A committee composed of 16 experts from four scientific societies, SFAR, SRLF, SFTS and SFVTT, evaluated three fields: (1) anemia prevention, (2) transfusion strategies and (3) non-transfusion treatment of anemia. Population, Intervention, Comparison, and Outcome (PICO) questions were reviewed and updated as needed, and evidence profiles were generated. Analysis of the literature and formulation of recommendations were then conducted according to the GRADE^®^ methodology.

**Results:**

The SFAR–SRLF guideline panel provided ten statements concerning the management of anemia in adult critical care patients. Acute haemorrhage and chronic anemia were excluded from the scope of these recommendations. After two rounds of discussion and various amendments, a strong consensus was reached for ten recommendations. Three of these recommendations had a high level of evidence (GRADE 1±) and four had a low level of evidence (GRADE 2±). No GRADE recommendation could be provided for two questions in the absence of strong consensus.

**Conclusions:**

The experts reached a substantial consensus for several strong recommendations for optimal patient management. The experts recommended phlebotomy reduction strategies, restrictive red blood cell transfusion and a single-unit transfusion policy, the use of red blood cells regardless of storage time, treatment of anaemic patients with erythropoietin, especially after trauma, in the absence of contraindications and avoidance of iron therapy (except in the context of erythropoietin therapy).

## Introduction

Anemia is very common in critical care patients, affecting about two-thirds of patients on admission, with a mean haemoglobin (Hb) level on admission of 11.0 g/dL [1, 2]. During the critical care stay, repeated blood loss (blood samples, invasive procedures, surgery, etc.), haemodilution and inflammation contribute to lowering haemoglobin concentration [3, 4]. The severity of anemia on admission is also associated with increased morbidity and mortality in critical care patients. Finally, this anemia can persist on the medium and long term, as more than one half of patients who were anaemic at the time of discharge from critical care were still anaemic 6 months after discharge [5]. In view of the high prevalence of anemia, a large proportion of critical care patients are exposed to blood transfusion [6]. Since the original publication by Hebert et al., which introduced the concept of a restrictive transfusion strategy in critical care [7], many studies have been conducted in this field and deserve to be specifically analysed in relation to critical care patients. The management of anemia in critical care patients therefore constitutes a challenge, but no guidelines concerning the prevention or treatment of anemia in this setting have been published.

**Fig. 1:**
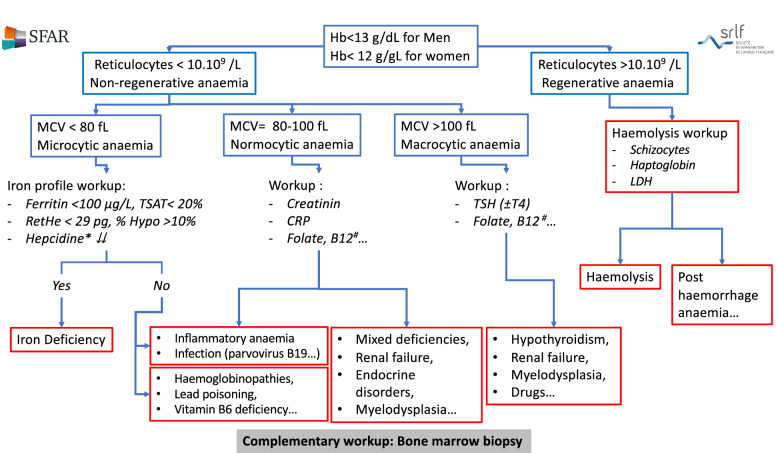
Anemia diagnostic flow-chart. This anemia diagnostic flow-chart is presented as a guide. Asterisk: Hepcidin is not yet available in routine clinical practice. Hash: WHO defines folate deficiency as serum folate < 10 nmol/L (4.4 g/L) or red blood cell folate, reflecting long-term status and tissue reserves, < 305 nmol/L (< 140 µg/L). Serum vitamin B12 < 150 pmol/L (< 203 ng/L) indicates vitamin B12 deficiency and a higher level does not exclude vitamin B12 deficiency, in which case blood methylmalonic acid must be assayed (a level > 271 nmol/L is in favour of vitamin B12 deficiency)

The management of anemia is based on a standardised diagnostic work-up (a diagnostic flow-chart is presented in Fig. 1). The main mechanisms of anemia in critical care are as follows:

- Blood loss, leading to loss of red blood cells and iron deficiency [8],
- Haemodilution,
- Inflammation, responsible for inhibition of endogenous erythropoietin (EPO) synthesis, inhibition of the bone marrow response to EPO and for functional iron deficiency due to induction of hepcidin synthesis [4, 9]. Inflammation is also responsible for decreased red blood cell life span, especially as a result of membrane alterations [10].

## Purpose of the guidelines

The purpose of these clinical practice guidelines (CPG) is to propose a framework to facilitate decision-making in anaemic patients, while also facilitating implementation of procedures designed to prevent the development of anemia in critical care patients. The expert group has strived to produce a minimal number of guidelines in order to highlight the key points in the three fields defined (see below). In doubtful situations, evidence from the literature took precedence over expert opinion. Basic good clinical practice in intensive care was considered to be already established and was excluded from these CPG. The management of chronic anemia and haemoglobinopathies, as well as the management of acute haemorrhagic anemia, already the subject of SFAR/SRLF/SFMU joint guidelines [11], were also excluded from these CPG. A large public is concerned by these guidelines, corresponding to all critical care professionals.

### Definition

Anemia is defined by the World Health Organization as a Hb concentration < 13.0 g/dL in men and < 12.0 g/dL in women.

## Method

### General organisation

These guidelines are the result of the work conducted by a SFAR and SRLF joint expert committee. Each expert completed a conflicts of interest declaration before starting the literature review. The expert committee agenda was defined at the beginning. The organisation committee initially defined the questions to be addressed in collaboration with the coordinators. Experts in charge of each question were then appointed. Questions were formulated according to a PICO (Patient Intervention Comparison Outcome) format after the first expert committee meeting. Review of the literature and formulation of recommendations were then conducted according to the GRADE methodology (Grade of Recommendation Assessment, Development and Evaluation). A level of evidence was defined for each publication cited as a function of the study design. This level of evidence could be revised by taking into account the methodological quality of the study. A global level of evidence was determined for each endpoint by taking into account the levels of evidence of each publication, the consistency of the results between the various studies, the direct or indirect nature of the evidence, and the cost analysis. A “high” global level of evidence permitted the formulation of a “strong” recommendation [“it is recommended to…” (GRADE 1+) or “it is not recommended to…” (GRADE 1−)]. When the global level of evidence was moderate, low or very low, an optional recommendation was formulated [“it is probably recommended to…” (GRADE 2+) or “it is probably not recommended to…” (GRADE 2−)]. When the literature was non-existent or insufficient, the recommendation concerning the question was based on expert opinion (“the experts suggest…”). Proposed recommendations were presented and discussed one by one. The purpose of this process was not to inevitably reach a unique, convergent expert agreement on all proposals, but to define points of concordance, divergence or indecision. Each recommendation was then evaluated by each expert, who provided an individual score using a scale ranging from 1 (complete disagreement) to 9 (complete agreement). The collective score was established according to a GRADE grid methodology. In order to validate a recommendation according to a particular criterion, at least 50% of experts had to express an opinion globally in favour of the recommendation, while less than 20% of experts expressed an opposite opinion. To obtain a strong recommendation, 70% of experts had to agree with the recommendation. In the absence of agreement, the recommendations were reformulated and rescored in order to reach an agreement.

### Scope of guidelines

Three fields were defined as follows: prevention of anemia, transfusion strategies and treatment of anemia other than by transfusion. As indicated above, chronic anemia, haemoglobinopathies and acute blood loss anemia, as well as specific paediatric entities, were excluded. An extensive literature search over the last 25 years was performed on PubMed™ and Cochrane™ databases. To be included in the analysis, publications had to be written in French or English. It was decided, before starting the analysis, to limit the number of expert opinions and to only propose evidence-based recommendations. The literature review focused on recent data according to an order of assessment ranging from meta-analyses and randomised trials to observational studies. Sample sizes and the relevance of the research were considered for each study. Endpoints considered to be significant were mortality, critical care length of stay, need for or duration of organ support and use of transfusion. An increase of haemoglobin levels was not considered to be an objective per se.

These CPG replace the previous guidelines on the same topic issued by SFAR and the SRLF. Both of them encourage all critical care physicians to comply with these guidelines to ensure optimal quality of patient care. However, each physician must exercise his/her own judgement in the application of these guidelines, taking into account his/her experience and the specificities of his/her institution, to determine the intervention best adapted to the patient’s condition.

## Summary of the results

Analysis of the literature by the experts and application of the GRADE methodology with two scoring rounds resulted in the proposal of ten recommendations with a strong consensus and a summary table indicating target Hb in the case of transfusion. Three of the ten formal recommendations have a high level of evidence (GRADE 1±), four have a low level of evidence (GRADE 2±) and three are based an expert opinion. The indicative summary table of target Hb is also based on expert opinion. No recommendations could be proposed for two PICO questions: (1) the transfusion threshold in critical care patients with chronic cardiovascular disease (see rationale for recommendation R2.1); (2) administration of vitamin B12 and/or folic acid to critical care patients (see recommendation R3.4).

## Field 1. Which non-pharmacological interventions can reduce red blood cell transfusion and/or morbidity and mortality related to anemia or transfusion in critical care patients?

Expert: Olivier Huet

**Table:** 

R1.1—The experts propose a diagnostic phlebotomy reduction strategy (volume and number) to decrease the incidence of anemia and transfusion in critical care patients.
EXPERT OPINION, STRONG AGREEMENT

### Rationale

Diagnostic phlebotomy is performed very frequently in critical care patients, with a mean daily volume of about 40–80 mL. These iatrogenic blood losses contribute to the development of anemia in critical care patients [12]. Phlebotomy tubes are also frequently flushed, resulting in considerable blood loss. The non-pharmacological prevention of anemia in critical care patients consists of interventions designed to decrease these blood losses.

The main non-pharmacological interventions designed to reduce the risk of anemia are: blood test reduction strategies, as already proposed in the SFAR–SRLF joint CPG on the relevance of blood tests and chest X-rays in intensive care [13], reduction of the blood volumes drawn and use of blood conservation systems after drawing blood from an arterial catheter.

A single-centre randomised trial on blood test reduction strategies, with a high risk of bias [14], reported a significant reduction of the blood volume drawn [8 (interquartile range: 7–10) versus 40 (28–43) mL/day, *p* < 0.001] with no impact on the patients’ Hb concentrations.

Most studies on reduction of the blood volumes drawn are observational [15–18]. Phlebotomy devices appear to decrease the volume of blood drawn, but the volume saved cannot be evaluated due to the heterogeneity of the studies and their methodological bias.

In prospective randomised trials, the use of blood conservation devices appears to decrease the blood volume drawn [19, 20], but only one study reported a significant reduction of transfusion requirements [19]. However, these studies present major methodological biases.

A systematic review of the recent literature summarised the state of knowledge concerning the impact of phlebotomy reduction and blood conservation devices after drawing blood from an arterial catheter on the morbidity and mortality related to anemia [21]. It showed that strategies based on the use of paediatric tubes allow a 29 to 74% reduction of the blood volume drawn, depending on the study, and that devices designed to conserve blood flushed from arterial catheters allow a 19 to 80% reduction of the blood volume drawn. A combination of these various interventions could be beneficial [14].

## Field 2: Which transfusion strategies can reduce red blood cell transfusion and/or morbidity and mortality related to anemia in critical care patients?

Experts: Cécile Aubron, Sylvain Ausset, Pierre Buffet, Hafid Ait-Oufella, Yoann Launey, Hervé Quintard

**Table:** 

R2.1—It is recommended to adopt a restrictive transfusion strategy (Hb threshold: 7.0 g/dL) in critical care patients in general, including septic patients, in order to reduce the use of red blood cell transfusion without increasing morbidity and mortality.
(GRADE 1+), (STRONG AGREEMENT)

### Rationale

The pioneer TRICC trial (Transfusion Requirements In Critical Care) by Hebert et al. including 838 critical care patients with normovolaemic anemia did not reveal any significant difference in terms of 30-day mortality between a restrictive transfusion strategy (single-unit transfusion to a transfusion threshold of 7.0 g/dL of Hb for a Hb target between 7.0 and 9.0 g/dL) and a liberal transfusion strategy (single-unit transfusion to a transfusion threshold of 10.0 g/dL of Hb for a target Hb between 10.0 and 12.0 g/dL) [7]. A significant reduction of the number of units of red blood cells transfused was observed in favour of the restrictive strategy (2.6 ± 4.1 versus 5.6 ± 5.3 units of red blood cells transfused, *p *< 0.01).

A large randomised trial, Transfusion Requirements In Septic Shock (TRISS), comparing Hb transfusion thresholds of 7.0 g/dL and 9.0 g/dL in patients with septic shock did not reveal any significant difference in terms of 90-day mortality between patients receiving either of these two transfusion strategies [216/502 (43%) versus 223/496 (44.9%)] [22]. A similar rate of ischaemic events was observed in the two arms. Red blood cell transfusion was performed significantly less often in the restrictive transfusion strategy arm than in the liberal transfusion strategy arm (median of one unit versus four units, *p* < 0.001). An ancillary study of the TRISS trial did not reveal any significant difference in terms of 1-year mortality (53.3% versus 54.6%) [23]. Another post hoc analysis of the TRISS trial, based on the new definition of septic shock, did not reveal any significant difference in mortality [135/275 (49%) versus 151/279 (54%)] [24]. The TRISS trial applied a transfusion strategy throughout the hospitalisation of patients managed for septic shock, while other studies were more specifically devoted to the initial management (first 72 h of septic shock). The pivotal study by Rivers et al. suggested a benefit of maintaining haematocrit at 30% (Hb ≈ 10.0 g/dL) at the initial phase of management of patients with severe sepsis in the context of the “early goal-directed therapy” resuscitation protocol [25]. It should be stressed that two-thirds of the patients included in the interventional arm of this single-centre study had therefore received red blood cell transfusion during the first 6 h of management. A decade later, the three trials replicating this resuscitation strategy (PROMISE, PROCESS, ARISE) evaluated the benefit of a combination of measures applied at the initial phase of management to achieve a target of ScvO_2_ ≥ 70%, including blood transfusion to maintain haematocrit > 30% or Hb > 10.0 g/dL [26–28]. However, only the control arm of the PROCESS trial explicitly proposed a restrictive transfusion Hb threshold of 7.5 g/dL [28]. These three trials concluded on the absence of survival difference between interventional and control arms. However, fewer than 15% of patients received blood transfusions during the first 6 h.

Finally, two single-centre trials published by the same Brazilian team [29, 30] compared restrictive (Hb threshold of 7.0 g/dL) and liberal (Hb threshold of 9.0 g/dL) transfusion strategies in the specific population of cancer patients admitted to critical care postoperatively after major abdominal surgery or for septic shock. These trials showed a trend towards lower mortality in the liberal transfusion strategy arms. However, these trials present a risk of bias and their limited sample sizes were not sufficient to modify the conclusions of meta-analyses or to challenge the general principle of restrictive transfusion.

It must be stressed that the decision of whether or not to transfuse a patient must not be exclusively based on the Hb level, but must take into account the patient’s tolerance of anemia, particularly in patients with cardiovascular disease.

Data specifically concerning the transfusion threshold in critical care patients with chronic cardiovascular disease present a low level of evidence. This patient population could potentially have a coronary network that is more sensitive to limitation of the oxygen supply. A meta-analysis published in 2016, based on 11 randomised trials including 3033 patients, assessed the impact of the transfusion strategy on 30-day morbidity and mortality in patients with cardiovascular diseases [31]. Restrictive transfusion strategies (Hb thresholds generally between 7.0 and 8.0 g/dL) were not inferior to liberal strategies (Hb thresholds generally between 9.0 and 10.0 g/dL) in terms of 30-day mortality, but a higher risk of acute coronary syndrome was observed in the restrictive transfusion arm [RR 1.78 95% confidence interval (1.18–2.70)]. The main limitations of this meta-analysis were the heterogeneity of the populations included in the trials and their sometimes small sample sizes, including critical care patients, but also patients managed for hip fracture and finally, patients with pre-existing coronary artery disease, as well as acute coronary syndromes. Furthermore, some randomised trials including patients with pre-existing coronary artery disease were not included in the meta-analysis. The methods used to diagnose cardiac events also varied considerably between trials (detection bias). In an attempt to make this population more homogeneous, we conducted a new meta-analysis exclusively targeting critical care patients with known chronic cardiovascular disease (354 patients in the restrictive transfusion strategy arm and 376 in the liberal transfusion strategy arm). We did not observe any significant difference in terms of mortality or new-onset acute coronary syndrome between the two transfusion strategies, suggesting that an Hb threshold of 7.0 g/dL is sufficient.

A strong consensus could not be reached by the experts concerning the proposal of a recommendation to adopt a restrictive threshold (Hb: 7.0 g/dL) in critical care patients with chronic cardiovascular disease. This persistent uncertainty justifies new more homogeneous randomised trials in this patient population.

**Fig. 2:**
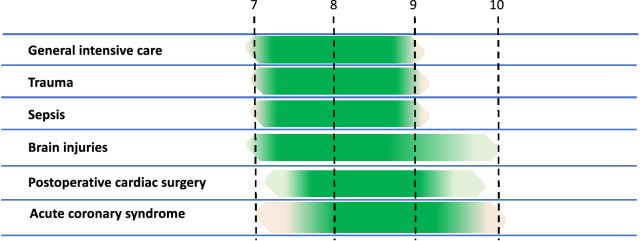
Target haemoglobin in the case of transfusion (Expert opinion). The following figure proposes target Hb levels for transfusion in critical care patients, as a function of various clinical settings (i.e. the haemoglobin level below which (lower bound) single-unit transfusion is probably recommended to achieve Hb not exceeding the upper bound). The shaded zones on the figure represent the degree of uncertainty according to the experts, which is why this figure is proposed on the basis of expert opinion. The GRADE level of recommendation is indicated for each setting, in accordance with the above recommendations (R2.1 to R2.4). Note that these targets apply in the absence of active bleeding or poorly tolerated anemia, especially with cardiovascular symptoms

Figure 2 proposes transfusion thresholds adapted to the various populations of critical care patients (Expert opinion). *Strong consensus*.

**Table:** 

R2.2—It is recommended to adopt a restrictive transfusion strategy (Hb threshold between 7.5 and 8.0 g/dL) in postoperative cardiac surgery critical care patients in order to reduce the red blood cell transfusion rate without increasing morbidity and mortality.
(GRADE 1+), STRONG AGREEMENT

### Rationale

Three large-scale randomised controlled trials have evaluated transfusion thresholds in elective cardiac surgery [32–34]. Two recent meta-analyses [35, 36] of randomised controlled trials, including 8838 and 8886 patients, and a subgroup analysis of another meta-analysis [37] including 7441 patients, demonstrated no significant difference in terms of 30-day mortality between the restrictive transfusion strategy arms (Hb thresholds ranging from 7.5 to 8.0 g/dL) and the liberal transfusion strategy arms (Hb thresholds ranging from 9.0 to 10.0 g/dL). The non-inferiority of a restrictive transfusion strategy also persisted on analysis of 6-month mortality [38]. In these meta-analyses, the number of units of red blood cells transfused per patient was significantly lower in the restrictive transfusion strategy arm and no significant difference in terms of adverse events, including myocardial infarction, arrhythmias, stroke, acute renal failure or infections, was observed between the two arms. Restrictive transfusion strategies reduce the use of blood products without increasing morbidity and mortality in postoperative cardiac surgery critical care patients.

**Table:** 

R2.3—It is probably not recommended to adopt a liberal transfusion strategy targeting Hb > 10.0 g/dL in order to decrease the morbidity and mortality in patients with revascularised or non-revascularised acute coronary syndrome.
(GRADE 2−), STRONG AGREEMENT

### Rationale

Several retrospective studies have reported an association between red blood cell transfusion and an excess risk of mortality and cardiovascular events (re-infarction, heart failure, stroke). These results were confirmed by a recent meta-analysis [39], in which transfusion was associated with a non-significant reduction of mortality when Hb was less than 8.0 g/dL [OR 0.52 (0.25–1.06)], but was associated with increased mortality when Hb was greater than 10.0 g/dL (OR 3.34 (2.25–4.97). However, this meta-analysis was based on retrospective studies comprising a number of confounding factors (age, comorbidities, bleeding).

Only two randomised interventional trials have been published. Cooper et al. compared a restrictive strategy (haematocrit 24–27%) to a liberal strategy (haematocrit > 30%) [40]. The liberal strategy was associated with an increase of the composite endpoint (mortality, recurrent myocardial infarction, episode of heart failure) 38% versus 13% (*p* = 0.046). However, only 45 patients were included over a period of 6 years. Carson et al. compared a restrictive strategy (Hb > 8.0 g/dL) versus a liberal strategy (Hb > 10.0 g/dL) [41]. A total of 110 patients were included in 8 centres. The liberal strategy was associated with a non-significant reduction of the composite endpoint (mortality, infarction, unscheduled coronary revascularisation up to 30 days after randomisation) (10.9% versus 25.9%, *p* = 0.054) and a significant reduction of 30-day mortality (1.8% versus 13%, *p* = 0.032). Ongoing prospective randomised trials should help to define transfusion thresholds in these populations (especially the REALITY trial, NCT02648113).

**Table:** 

R2.4—It is probably not recommended to adopt a liberal transfusion strategy targeting Hb > 10.0 g/dL to decrease morbidity and mortality in brain injured patients.
(GRADE 2−), STRONG AGREEMENT

### Rationale

A review of the literature conducted in 2012 and including six trials and 537 patients, with four trials in traumatic brain injury patients, one trial in patients with meningeal haemorrhage and one trial on a mixed population, compared low transfusion thresholds (Hb 7.0–10.0 g/dL) to high Hb thresholds (Hb 9.3–11.5 g/dL) [42]. This review did not reveal any significant difference in terms of mortality between the two strategies, but reported a shorter length of hospital stay in the restrictive transfusion strategy arm. A retrospective study in 215 traumatic brain injury patients reported increased mortality, a higher rate of neurological complications, and a longer hospital stay in transfused patients [43]. These trials were very heterogeneous and no effect on overall mortality was detected, but they provide arguments against transfusion in traumatic brain injury patients (prolonged stay, vasospasm, thrombosis, neurological composite endpoint, etc.) that may justify the recommendation of low transfusion thresholds. A prospective randomised trial with a 2 × 2 factorial plan evaluating both transfusion thresholds and adjuvant erythropoietin therapy did not reveal any significant difference in terms of morbidity and mortality, but demonstrated decreased transfusion requirements in the restrictive transfusion arm [44]. Several randomised trials are currently underway and may help to define the transfusion strategy in this population (HEMOTION trial, NCT03260478; TRAIN trial, NCT02968654).

**Table:** 

R2.5—It is not recommended to select units of red blood cells according to their duration of storage to decrease the morbidity and mortality in critical care patients.
(GRADE 1−), STRONG AGREEMENT

### Rationale

The maximum duration of storage of red blood cells allowed in France is 42 days. When several compatible units of red blood cells are available for transfusion, standard procedure consists of delivering the oldest unit in order to avoid wasting precious labile blood products and to ensure optimal stock management. Red blood cells undergo certain changes during storage, affecting both erythrocytes and the storage medium. These changes are described as “storage lesions” [45]. In vitro, experimental and observational studies, including the pioneer critical care studies based on relatively small sample sizes, suggest a harmful effect associated with the duration of red blood cell storage [46, 47]. Two large-scale randomised controlled trials, ABLE (2430 patients) and TRANSFUSE (4828 patients), have been conducted in critical care patients and did not demonstrate any impact of red blood cell storage on outcomes [48, 49]. Two recent meta-analyses were based on 16 randomised trials in various adult and paediatric medical and surgical populations and seven randomised trials in critical care patients, respectively [50, 51]. In these trials, the use of fresh red blood cells, i.e. generally less than eight days old and almost always less than 12 days old, was not associated with any significant benefit in terms of early or late mortality (up to 90 days for critical care patients), transfusion-related adverse effects or the incidence of post-transfusion nosocomial infections. These conclusions remain valid in critical care and cardiac surgery subpopulations. The currently available results therefore do not call into question the standard procedure concerning the choice of unit of red blood cells.

**Table:** 

R2.6—The experts suggest adoption of a restrictive transfusion strategy based on transfusion of a single unit of red blood cells followed by review of the indication for subsequent transfusion in order to reduce red blood cell utilisation without increasing morbidity and mortality.
EXPERT OPINION, STRONG AGREEMENT

### Rationale

To our knowledge, no randomised trial has compared single-unit red blood cell transfusion with multiple-unit transfusion in haemodynamically stable anaemic patients. Single-unit transfusion constitutes part of restrictive transfusion strategies and is a cornerstone of patient blood management (PBM). Despite the absence of a high level of evidence, single-unit transfusion is included in the majority of guidelines [52]. The benefit of this type of transfusion practice is supported by a number of factors. Firstly, single-unit transfusion is not associated with an excess risk in haemodynamically stable anaemic patients. Secondly, single-unit transfusion is associated with a reduction of the number of units of red blood cells transfused.

The pioneer TRICC trial was the first large-scale randomised trial to have applied single-unit transfusion [7]. All patients of this study received one unit of red blood cells, followed by review of the indication for transfusion. The other randomised trials that have evaluated transfusion thresholds also applied single-unit transfusion to all patients. Although these trials were unable to demonstrate the benefit of single-unit transfusion, they nevertheless support the absence of excess risk associated with this type of transfusion strategy.

Several observational studies have reported the impact of single-unit transfusion on the number of units of red blood cells transfused per transfusion episode and/or per patient. In an observational study conducted in critical care haematology patients, single-unit transfusion (applied to 126 patients) was compared to transfusion of two units of red blood cells (applied to 186 patients). Single-unit transfusion was associated with a reduction of the number of units of red blood cells administered to allogeneic stem cell transplant recipients (5.0 versus 7.7 units, *p* < 0.01). No difference in terms of morbidity and mortality was observed between the two arms [53]. In another cohort study of haematological oncology patients, single-unit transfusion versus transfusion of two units of red blood cells was independently associated with a reduction of 2.7 units of red blood cells per chemotherapy cycle [54].

Single-unit transfusion is also a key element of PBM programmes. In a multicentre trial studying the impact of a PBM programme, single-unit transfusion was applied to 70.9% of patients after implementation of the programme (versus 38% before implementation) and was an independent factor associated with a reduction of red blood cell utilisation [55]. Oliver et al. compared transfusion indications between two 6-month periods (before and after implementation of a PBM programme) and reported that single-unit transfusion was the key element associated with the reduction from 2 to 1.5 units of red blood cells per transfusion episode (*p* < 0.0001) [56].

## Field 3: In critical care patients, which non-transfusional treatments are able to reduce red blood cell transfusion and/or morbidity and mortality related to anemia or transfusion?

Experts: Matthieu Legrand, Thomas Lescot, Armand Mekontso Dessap, Michael Piagnerelli

### Question 1: Does administration of erythropoiesis-stimulating agents (ESA) reduce red blood cell utilisation and/or morbidity and mortality related to anemia or transfusion?

**Table:** 

R3.1—It is probably recommended to use erythropoiesis-stimulating agents in critically ill anaemic (Hb ≤ 10.0–12.0 g/dL) and/or trauma patients in the absence of contraindication, especially with a history of ischaemic cardiovascular disease and/or venous thromboembolism, in order to reduce red blood cell utilisation and decrease mortality.
(GRADE 2+), STRONG AGREEMENT

### Rationale

Several meta-analyses [57–61] have evaluated the use of erythropoiesis-stimulating agents (ESA) in critical care patients. The largest meta-analysis, which included 34 studies (25 randomised controlled trials and 9 observational studies with a total of 930,470 critical care patients), suggested a positive impact of ESA administration on mortality [RR 0.76, (0.61–0.92)] [58]. This meta-analysis confirmed the results of the meta-analysis conducted by French et al. that included nine randomised controlled trials, but only seven of which were double-blinded, with a total of 2607 critically ill trauma patients, which also showed that administration of ESA was associated with decreased mortality [RR 0.63 (0.49–0.79)] [57]. Note that only the meta-analysis by Zarychanski et al., based on seven randomised controlled trials, evaluated the impact of ESA on red blood cell requirements and reported a reduction of red blood cell utilisation with an RR of 0.73 (0.64–0.84) [60].

However, the authors of these meta-analyses downgraded the GRADE score in view of the high risk of bias, the inconsistency and the imprecision of the studies. In the meta-analysis by Mesgarpour [59], the randomised controlled trials included 10 trials conducted in critical care patients designed to treat anemia with a potential effect on mortality [59, 62–69], 3 trials in traumatic brain injury patients [70–72] and 13 trials in which ESA was administered as adjuvant therapy for ST segment elevation myocardial infarction. Variable weekly treatment regimens, intravenous and/or subcutaneous routes of administration, and timing of the start of treatment made the results difficult to interpret. The primary endpoint ranged between 5- and 30-day mortality after critical care admission. Complications (including deep venous thromboses) did not appear to be more frequent in the ESA arm, but these potential adverse effects were not systematically investigated and reported. The authors were therefore unable to reach an*y* conclusions in view of the heterogeneity of the studies included (*I*^2^ = 55% in the meta-analysis by Zarychanski et al. [61]). No data on disease progression in cancer patients treated by ESA were available.

In view of this uncertainty, ESA therapy should therefore be reserved to the patients most likely to benefit from this treatment (anaemic and/or trauma patients). In these subgroups of anaemic and/or trauma patients, ESA had a major impact on mortality, and the benefit-risk balance was probably favourable, especially in patients with a longer stay (more than 5 days).

The dose most commonly used in these studies was 40,000 IU by subcutaneous injection once weekly in combination with an iron supplement (oral or by injection when oral treatment was poorly tolerated, in the case of insufficient response or iron deficiency, generally defined in these trials as a transferrin saturation < 20% and/or a ferritin < 100 µg/L) and Hb threshold for inclusion was < 12.0 g/dL [63, 64]. It is therefore probably legitimate to propose these treatments to patients with Hb ≤ 10.0–12.0 g/dL.

**Table:** 

R3.2—The experts suggest stopping erythropoiesis-stimulating agents when haemoglobin stabilises between 10.0 and 12.0 g/dL in order to decrease morbidity and mortality.
EXPERT OPINION, STRONG AGREEMENT

### Rationale

In the majority of randomised controlled trials evaluating ESA in critical care patients, administration of ESA was stopped when Hb exceeded the threshold of 12.0 g/dL [63, 64, 67]. A meta-analysis of nine trials including 5143 non-critical care chronic kidney disease patients treated by ESA showed a higher mortality when a high Hb target (≥ 12.0 g/dL) was used compared to lower Hb targets (10.0–12.0 g/dL) [RR: 1.17 (1.01–1.35] [73]. In this meta-analysis, the use of high targets was also associated with an increased risk of arteriovenous access thrombosis [RR 1.34 (1.16–1.54)].

### Question 2. Should iron be administered to critical care patients to decrease red blood cell utilisation, morbidity and mortality?

**Table:** 

R3.3—It is probably not recommended to administer iron to reduce red blood cell utilisation or morbidity and mortality in critical care patients, except in combination with erythropoiesis-stimulating agents.
(GRADE 2−), STRONG AGREEMENT

### Rationale

Many studies have demonstrated the efficacy of intravenous iron to significantly increase Hb in patients with anemia, generally iron deficiency anemia, with a time to maximum efficacy of three to 4 weeks in the non-critical care setting (for example, preoperatively before orthopaedic surgery). The majority of studies specifically concerning critical care patients included patients admitted for trauma or postoperatively and excluded septic patients. They evaluated systematic iron supplementation (in the presence or absence of anemia), but not the treatment of iron deficiency (i.e. patients were not included on the basis of a diagnosis of iron deficiency). In a recent meta-analysis [74] including six randomised placebo-controlled trials, intravenous (five trials) or oral (one trial) iron administration was not associated with a lower rate of blood transfusion during the hospital stay, but was associated with a higher Hb concentration on discharge from hospital. However, the clinical relevance of this increased Hb would appear to be very limited [+0.31 (0.04–0.59) g/dL)].

Oral iron appears to be less effective than intravenous iron in unselected populations, but very few data are available in critical care patients (two randomised trials evaluated oral iron *versus* no iron in a total of 305 patients (OR 0.82 (0.54–1.25) on the blood transfusion rate) [75]. Although one meta-analysis including all populations [76] suggested an increased infectious risk, an increased risk was not observed in critical care patients. Finally, a risk of anaphylactic reaction was described with the use of intravenous iron, with a reported incidence of 68 per 100,000 patients (57.8–78.7) for iron dextran and 24 per 100,000 patients (20.0–29.5) for iron without dextran. The lowest risk was reported with iron sucrose [77]. New molecules are associated with an even lower risk of adverse events. Due to the insufficient power of studies conducted in critical care patients (a maximum of 97 patients included in the study by Pieracci et al. [78]), no significant effects were observed on the length of critical care stay or mortality.

Note that most trials on the use of ESA also corrected iron deficiency or systematically administered iron. These trials also evaluated systematic iron supplementation and not correction of iron deficiency, which is difficult to diagnose in the critical care setting. The study by Pieracci et al. showed that oral iron was effective to reduce transfusion in patients with iron deficiency, but not in patients without iron deficiency [79]. However, no published study has evaluated treatment of iron deficiency.

### Question 3. Should vitamin B12 or folic acid be administered to critical care patients to decrease red blood cell utilisation, morbidity and mortality?

**Table:** 

R3.4—No recommendation could be formulated concerning administration of vitamins to critical care patients in order to reduce red blood cell transfusion and/or morbidity and mortality related to anemia or transfusion.
NO RECOMMENDATION

### Rationale

No data are available concerning administration of vitamin B12 to critical care patients (with selected endpoints). Two trials reported the effects of prophylactic folate supplementation in critical care patients [80, 81]. One trial reported a lower proportion of patients with folate deficiency after 7 days of treatment (plasma folate concentration < 2.7 ng/mL) among those who had received intravenous folate supplementation at a dose of 5 mg per day (0%, *n* = 22) or 50 mg per week (4%, *n* = 24) in comparison with patients not receiving folate supplementation (27%, *n* = 37). The proportion of patients with baseline folate deficiency differed between the two arms, making it difficult to evaluate the efficacy of the intervention. In another trial evaluating the effects of two intravenous folate supplementation strategies (0.5 mg/day versus a single dose of 50 mg) in critical care patients with acquired folate deficiency, the authors reported an increase of total plasma and red blood cell folate concentrations on day-11. The changes observed cannot be attributed solely to this intervention in the absence of a control arm.

However, the WHO Recommended Daily Allowances are 0.4 to 1 mg of folate per day and 2.4 g of vitamin B12 per day. WHO also defines folate deficiency as serum folate < 10 nmol/L (4.4 µg/L) or red blood cell folate, reflecting long-term status and tissue reserves, < 305 nmol/L (< 140 µg/L). Serum vitamin B12 < 150 pmol/L (< 203 ng/L) indicates vitamin B12 deficiency and a higher level does not exclude vitamin B12 deficiency, in which case blood methylmalonic acid must be assayed (a level > 271 nmol/L is in favour of vitamin B12 deficiency).

## Data Availability

Not applicable.
